# Association of three micro-RNA gene polymorphisms with the risk of cervical cancer: a meta-analysis and systematic review

**DOI:** 10.1186/s12957-021-02463-4

**Published:** 2021-12-16

**Authors:** Jingyu Xu, Junze Geng, Qiang Zhang, Yihua Fan, Zijun Qi, Tian Xia

**Affiliations:** 1 First Teaching Hospital of Tianjin University of Traditional Chinese Medicine, Tianjin, 300193 China; 2 National Clinical Research Center for Chinese Medicine Acupuncture and Moxibustion, Tianjin, 300193 China; 3 Department of Oncology, Army Medical Center of PLA, Chong Qing, 400042 China; 4 Tianjin University of Traditional Chinese Medicine, Tianjin, 300000 China

**Keywords:** Micro-RNA, Single nucleotide polymorphism, Cervical cancer, Meta-analysis

## Abstract

**Objective:**

Regulation of single nucleotide polymorphisms (SNP) in micro-RNA (miRNA) on the host cells may be one of the most important factors influencing the occurrence of cervical cancer based on the prevalence of HPV infection and the development of cervical cancer. In order to explore the contribution of miRNA polymorphism to the occurrence and development of cervical cancer, we conducted an analytical study.

**Methods:**

We selected the polymorphisms of three widely studied miRNAs (miRNA-146a rs2910164, miRNA-499 rs3746444, and miRNA-196a2 rs11614913). Then, we conducted a meta-analysis (for the first time) to investigate their susceptibility to cervical cancer. Case control studies on the correlation between these three miRNAs and cervical cancer susceptibility were investigated by searching on from Pubmed, The Cochrane Library, Embase, CBM, CNKI, Wanfang database, and VIP database. Basic characteristics were recorded and meta-analysis of the case studies was performed using the STATA 15.1 software.

**Results:**

The miRNA-146a rs2910164 mutation significantly reduced the risk of cervical cancer in both recessive model (OR = 0.804, 95% CI = 0.652-0.992, *P* = 0.042; CC vs. CG+GG) and allelic model (OR = 0.845, 95% CI = 0.721-0.991, *P* = 0.038; C vs. G). There was no significant correlation between miRNA-499 rs3746444 and the risk of cervical cancer. The miRNA-196a2 rs11614913 mutation was significantly associated with a reduced risk of cervical cancer in homozygous model (OR = 0.641, 95% CI = 0.447-0.919, *P* = 0.016; TT vs. CC), dominant model (OR = 0.795, 95% CI = 0.636-0.994, *P* = 0.045; CT+TT vs. CC), recessive model (OR = 0.698, 95% CI = 0.532-0.917, *P* = 0.01; TT vs. CC+CT), and allelic models (OR = 0.783, 95% CI = 0.643-0.954, *P* = 0.015, T vs. C).

**Conclusion:**

In summary, this meta-analysis shows that the mutant genotypes of miRNA-146a rs2910164 and miRNA-196a2 rs11614913 are associated with a reduced risk of cervical cancer. Therefore, they may be two gene regulatory points for the prevention of cervical cancer.

**Systematic review registration:**

PROSPERO registration number CRD42021270079.

**Supplementary Information:**

The online version contains supplementary material available at 10.1186/s12957-021-02463-4.

## Introduction

Cervical cancer is the second most common malignancy in women, whose incidence is second only to breast cancer [1]. About 500,000 new cases of cervical cancer occur globally each year, accounting for 5% of all new cancer cases. Every year, more than 260,000 women die of cervical cancer [2], accounting for 7.5% of all female cancer deaths. The number is increasing year by year, and the patient population is getting younger. Therefore, cervical cancer is an important disease endangering women’s health and life [3, 4]. Focusing on women’s health is an important step in human reproduction and development. At present, in the face of cervical cancer, we still focus on prevention. Hence, the factors affecting the occurrence and development of cervical cancer are worthy of our research and discussion.

It is well-known that human papillomavirus (HPV) is associated with the occurrence of cervical cancer. HPV is the major risk factor for cervical cancer [5]. Women infected with HPV can get rid of it by their own immune system, but there are still some women who develop cervical cancer due to persistent infection with high-risk HPV [6]. In the process of continuous infection with the high-risk HPV until the development of cervical cancer, these women are also affected by environmental, genetic, and other factors [7].

Many diseases have been found to be related to genes. Nowadays, the genetic changes of patients and their influence on the occurrence and development of diseases are the focus of research. MicroRNAs (miRNAs) are a class of non-coding single-stranded RNA molecules with a length of about 22 nucleotides that regulate various intracellular activities such as cell proliferation, apoptosis, and carcinogenesis [8–10]. Each miRNA can have multiple target genes, and several miRNAs can regulate the same gene. MicroRNAs have been reported to regulate about one-third of human genes [11]. The length of miRNA is usually shorter than the length of the encoding gene. Its small genetic changes may alter human miRNA expression and target selection [12]. MicroRNAs have been shown to be associated with cervical cancer [13]. Therefore, single nucleotide polymorphisms (SNPs) in miRNA genes may lead to abnormal gene expression by changing miRNA maturation and expression [14, 15]. This may be related to cervical cancer susceptibility. In the past few years, several studies have investigated the association between some miRNA polymorphisms and cervical cancer risk, but the results were different [16–23]. Therefore, three miRNA polymorphisms (miRNA-146a rs2910164, miRNA-499 rs3746444, and miRNA-196a2 rs11614913) associated with cervical cancer risk were selected for meta-analysis. The polymorphism sites and base pair locations of the above three miRNAs are shown in Table 1.

**Table 1:** Basic information of three miRNA polymorphisms

Gene name	dpSNP rs#ID	Location	Chromosome	Alleles	Ancestral allele	Functional consequence
miRNA-146a	rs2910164	Pre-miRNA	5:160485411	G/C	G	NCTV*
miRNA-499	rs3746444	Pre-miRNA	20:34990448	T/C	T	NCTV*
miRNA-196a2	rs11614913	Pre-miRNA	12:53991815	C/T	C	NCTV*

*Note*: *NCTV* non_coding_transcript_variant

## Materials and methods

### Research program

This protocol of systematic review and meta-analysis has been drafted under the guidance of the Preferred Reporting Items for Systematic Reviews and Meta-Analyses Protocols (PRISMA-P). The meta-analysis was based on the Preferred Reporting Items for Systematic Reviews and Meta-Analyses (PRISMA) report (Table S1) [24]. It has been shared on open science framework (OSF) (registration number: DOI 10.17605/OSF.IO/P38H2). Moreover, it has been registered on PROSPERO (registration number: CRD42021270079).

### Literature search and selection

We searched Pubmed, The Cochrane Library, Embase, CBM, CNKI, Wanfang Database, and VIP database; all of which were established until May 2021. The retrieval method adopted the combination of subject words and free words. The retrieval strategy took PubMed as an example, as shown in Table S2. We searched all miRNA associated with cervical cancer susceptibility, the retrieved articles were screened by inclusion and exclusion criteria. Finally, three miRNA were included in this study (including miRNA-146a, miRNA-499, and miRNA-196a2). Where necessary, we contacted the original study author by email or phone for the undetermined but important information required in this study.

Inclusion criteria: (1) The association between miRNA and the cervical cancer risk was assessed; (2) studies with relevant genotype OR allele frequency, information to calculate OR and 95% CI; (3) the subjects were humans; (4) the study was designed as a case control.

Exclusion criteria: (1) Specific information on genotype or allele frequency of miRNA was not provided in the literature; (2) the genotype frequency of the control group was not in Hardy-Weinberg equilibrium; (3) the relationship with cervical cancer is unclear or the number of studies does not meet the miRNA for systematic evaluation; (4) cervical abscess cell pathology in the control group showed cervical intraepithelial neoplasia (CIN); (5) literatures with less than 6 stars in the Newcastle-Ottawa Scale [25].

### Data extraction and quality evaluation

The final included literature recorded the following information: first author name, year of publication, patient nationality, genotyping method, genotype, and allele frequency, and tabulated the basic characteristics. The selected literature was evaluated using the Newcastle-Ottawa Scale.

### Statistical analysis

Meta-analysis was used to assess the relationship between three miRNA polymorphisms and cervical cancer susceptibility. The genotype frequency of the control group was tested by the Hardy-Weinberg equilibrium (HWE) test using the chi-square test. If *P* > 0.05, it was regarded to be in the Hardy-Weinberg equilibrium. The odds ratio (OR) and 95% confidence interval (95%CI) were calculated to evaluate the relationship between the six gene models (homozygous, heterozygous, dominant, recessive, over dominant, and allelic genetic models) of the polymorphisms of miRNA-146a rs2910164, miRNA-499 rs3746444, and miRNA-196a2 rs11614913 and the risk of cervical cancer. The strength of association between each polymorphism and the risk of cervical cancer was evaluated by the combined odds ratio (OR) and its 95% confidence interval (95% CI). The statistical significance of combined OR was tested by *z* test; and *P* < 0.05 showed a significant statistical difference. We assessed heterogeneity between studies by chi-square test and *I*^2^: If *P* ≥ 0.1 and *I*^*2*^ < 50%, we used a fixed-effect model to assess OR and 95% CI. If *P* < 0.1 and *I*^2^ > 50%, we evaluated OR and 95% CI using a random effect model.

This meta-analysis quantitatively assessed publication bias using the Begg’s test and the Egger’s test. Sensitivity analysis assessed the stability of the results by excluding each study in turn. All analyses were performed using the STATA 15.1 software (STATA Corporation, College Station, TX, USA).

## Results

### Literature screening results

**Fig. 1:**
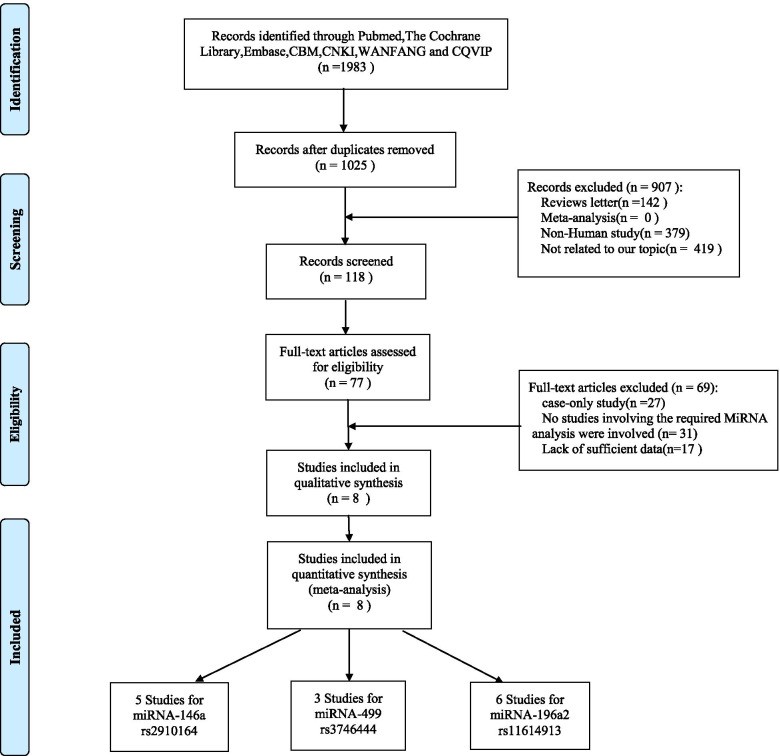
Flow diagram of study selection for this meta-analysis

In total, 8 articles were included in this meta-analysis [16–23]. From the studies, 14 data sets, comprising 3142 patients and 3971 controls were retrieved. Among these, five studies related to the correlation between miRNA-146a rs2910164 and cervical cancer; three related to the correlation between miRNA-499 rs3746444 and cervical cancer; and six related to the correlation between miRNA-196a2 rs11614913 and cervical cancer. The flowchart of literature screening is as shown in Fig. 1.

### Basic characteristics of the included literature and results of quality assessment

The basic characteristics of the literature are described in Table 2. Eight literatures were evaluated by Newcastle-Ottawa Scale, and the scores were all above 7☆ (full score was 9☆). The quality evaluation results of the included literature are as shown in Table 3.

**Table 2:** Characteristics of the studies eligible for meta-analysis

Frist author	Year	Country	Source of control	Genotyping method	Case/control	Case					Control					HWE*
MiRNA-146a rs2910164						CC	CG	GG	C	G	CC	CG	GG	C	G	
Bin Zhou	2011	China	HB*	PCR-RFLP*	226/309	70	113	43	253	199	116	159	34	391	227	0.060
Cong Yue	2011	China	HB*	PCR-RFLP*	447/443	105	224	118	434	460	150	206	87	506	380	0.285
Li Ma	2015	China	HB*	PCR-RFLP*	205/415	53	102	50	208	202	103	219	93	425	405	0.254
ShiZhi Wang	2019	China	HB*	TaqMan	954/1339	318	475	141	1111	757	471	631	212	1573	1055	0.978
Nisha Thakur	2019	India	HB*	PCR-RFLP*	150/150	21	49	80	91	209	28	49	73	105	195	0.056
MiRNA-499 rs3746444						TT	CT	CC	T	C	TT	CT	CC	T	C	
Bin Zhou	2011	China	HB*	PCR-RFLP*	226/309	134	84	8	352	100	223	71	15	517	101	0.051
ShiZhi Wang	2019	China	HB*	TaqMan	954/1339	675	228	27	1578	282	946	339	35	2231	409	0.485
Nisha Thakur	2019	India	HB*	PCR-RFLP*	150/150	25	47	78	97	203	21	49	80	91	209	0.063
MiRNA-196a2 rs11614913						TT	CT	CC	T	C	TT	CT	CC	T	C	
Bin Zhou	2011	China	HB*	PCR-RFLP*	226/309	57	123	46	237	215	82	169	58	333	285	0.077
Bo Ding	2016	China	HB*	TaqMan*	509/562	133	265	111	531	487	181	278	103	640	484	0.836
ChunTao Wang	2016	China	HB*	PCR-LDR*	104/186	31	52	21	114	94	65	82	39	212	160	0.170
ZhiLing Yan	2019	China	HB*	TaqMan	547/567	117	277	153	511	583	153	282	132	588	546	0.926
ShiZhi Wang	2019	China	HB*	TaqMan	954/1339	271	464	194	1006	852	424	629	269	1477	1167	0.201
Nisha Thakur	2019	India	HB*	PCR-RFLP*	150/150	17	58	75	92	208	57	51	42	165	135	0.089

*Note*: *HWE* Hardy-Weinberg equilibrium, *HB* hospital-based, *PCR-RFLP* restriction fragment length polymorphism polymerase chain reaction, *PCR-LDR* polymerase chain reaction-ligase detection reaction

**Table 3:** Newcastle-Ottawa Scale for quality assessment

Author (year)	Selection				Comparability	Exposure			Total score
	Case definition	Case representation	Selection of controls	Definition of contrast	Control for factor	Determination of exposure	Same exposure method	No response rates	
Zhou (2011) [16]	☆	☆		☆	☆☆	☆	☆	☆	8
Yue (2011) [17]	☆	☆		☆	☆☆	☆	☆	☆	8
Ma (2015) [18]	☆	☆		☆	☆	☆	☆	☆	7
Ding (2016) [19]	☆	☆	☆	☆	☆☆	☆	☆	☆	9
Wang (2016) [20]	☆	☆		☆	☆☆	☆	☆	☆	8
Yan (2019) [21]	☆	☆		☆	☆	☆	☆	☆	7
Wang (2019) [22]	☆	☆		☆	☆	☆	☆	☆	7
Thakur (2019) [23]	☆	☆	☆	☆	☆☆	☆	☆	☆	9

☆The article scored one point in the project

☆☆The article scored two points in the project

### Meta-analysis of the relationship between three miRNAs and cervical cancer

#### Relationship between miRNA-146a rs2910164 and cervical cancer

**Fig. 2:**
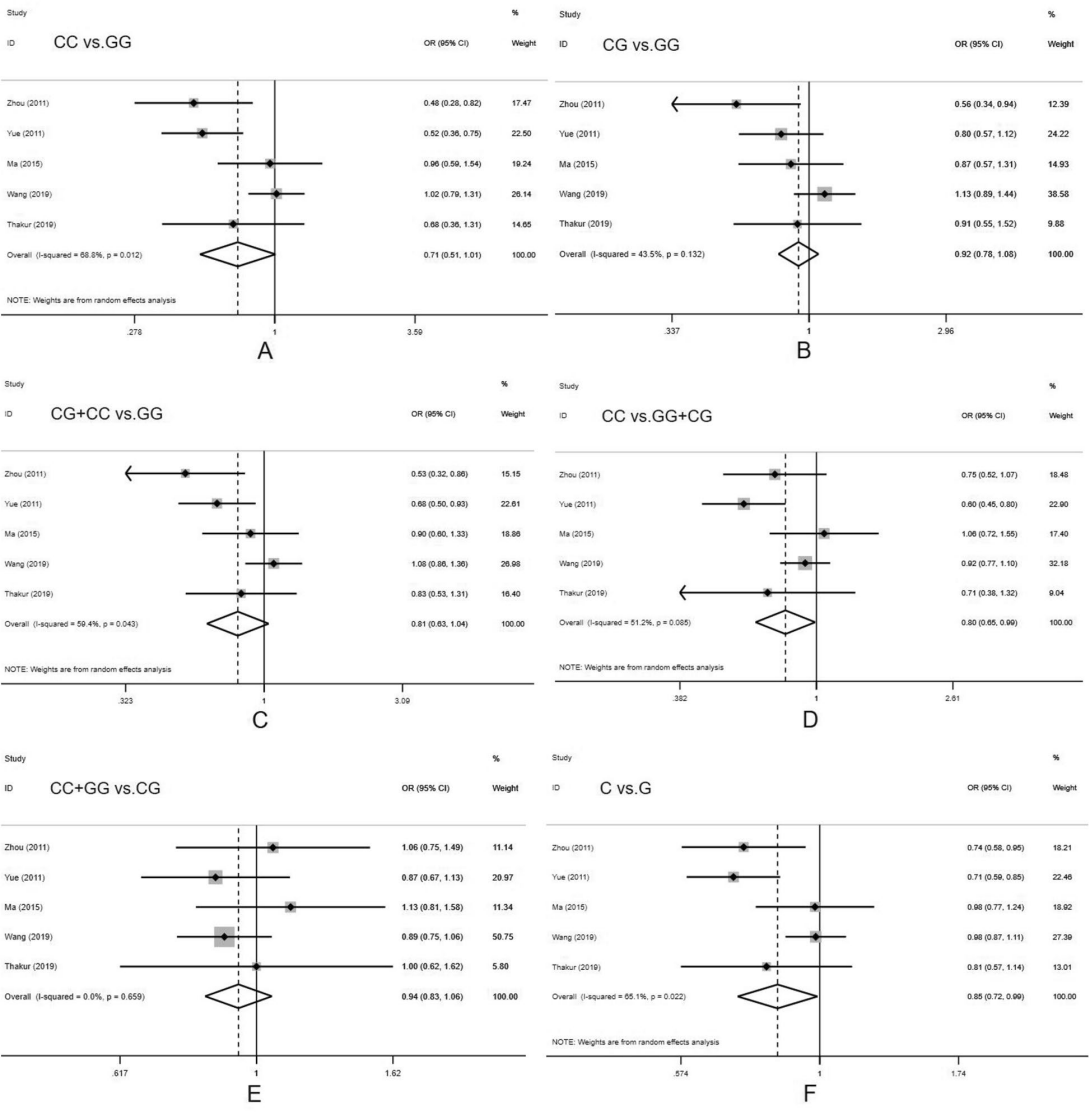
Forest plot for the association between miRNA-146a rs2910164 polymorphism and cervical cancer susceptibility for CC vs. GG (**A**), CG vs. GG (**B**), CG+CC vs. GG (**C**), CC vs. CG+GG (**D**), CC+GG vs. CG (**E**), and C vs. G (**F**)

The combined analysis showed that the mutant C allele significantly reduced the risk of cervical cancer in both the recessive genetic model (OR = 0.804, 95% CI = 0.652-0.992, *P* = 0.042; CC vs. CG+GG) and the allele genetic model (OR = 0.845, 95% CI = 0.721-0.991, *P* = 0.038; C vs. G), and the difference was statistically significant (Table 4 and Fig. 2).

**Table 4:** Total OR and 95% CI of three MiRNA polymorphisms in relation to cervical cancer susceptibility

Polymorphism	*N*	Genetic model	Association text			Heterogeneity text		
			OR [95% CI]	*z*	*p*	*X*^2^	*I*^2^ (%)	*p*
MiRNA-146a rs2910164	5	CC vs. GG	0.713 [0.505-1.006]	1.93	0.054	12.84	68.8	0.012
		CG vs. GG	0.920 [0.790-1.080]	1.03	0.304	7.08	43.5	0.132
		CG+CC vs. GG	0.807 [0.627-1.038]	1.67	0.095	9.84	59.4	0.043
		CC vs. GG+CG	**0.804 [0.652-0.992]**	2.04	0.042	8.2	51.2	0.085
		CC+GG vs. CG	0.939 [0.834-1.056]	1.05	0.293	2.42	0	0.659
		C vs. G	**0.845 [0.721-0.991]**	2.07	0.038	11.46	65.1	0.022
MiRNA-499 rs3746444	3	CC vs. TT	0.958 [0.663-1.383]	0.23	0.818	0.46	0	0.794
		CT vs. TT	1.173 [0.678-2.032]	0.57	0.568	12.04	83.4	0.002
		CT+CC vs. TT	1.140 [0.719-1.809]	0.56	0.577	9.65	79.3	0.008
		CC vs. CT+TT	0.966 [0.705-1.324]	0.22	0.829	0.68	0	0.71
		TT+CC vs. CT	0.833 [0.511-1.358]	0.73	0.464	12.2	83.6	0.002
		C vs. T	1.079 [0.832-1.401]	0.57	0.566	5.71	65	0.058
MiRNA-196a2 rs11614913	6	TT vs.CC	**0.641 [0.447-0.919]**	2.42	0.016	22.99	78.2	< 0.001
		CT vs. CC	0.922 [0.804-1.056]	1.17	0.24	3.68	0	0.597
		CT+TT vs. CC	**0.795 [0.636-0.994]**	2.01	0.045	12.71	60.7	0.026
		TT vs. CT+CC	**0.698 [0.532-0.917]**	2.58	0.01	20.93	76.1	0.001
		TT+CC vs. CT	0.917 [0.824-1.020]	1.6	0.11	1.12	0	0.952
		T vs. C	**0.783 [0.643-0.954]**	2.43	0.015	28.59	82.5	< 0.001

Subgroup analysis was performed according to the analysis methods used for genotyping and countries. In the study involving PCR-RFLP analysis, the polymorphism of miRNA-146a rs2910164 reduced the risk of cervical cancer in the homozygote (OR = 0.626, 95% CI = 0.453-0.865, *P* = 0.004; CC vs. GG), heterozygote (OR = 0.787, 95% CI = 0.637-0.972, *P* = 0.026; CG vs. GG), dominant (OR = 0.726, 95% CI = 0.590-0.893, *P* = 0.002; CC+CG vs. GG), recessive (OR = 0.753, 95% CI = 0.581-0.976, *P* = 0.032; CC vs. CG+GG), allele (OR = 0.795, 95% CI = 0.681-0.928, *P* = 0.004; C vs. G) genetic models, and the difference was statistically significant. The difference was not statistically significant in the analysis of China and India (Table 5).

**Table 5:** A subgroup analysis of the relationship between three MiRNA polymorphisms and susceptibility to cervical cancer

Variable	No.	CC vs. GG		CG vs. GG		CG+CC vs. GG		CC vs. GG+CG		CC+GG vs. CG		C vs. G	
MiRNA-146a rs2910164		OR [95% CI]	*P*	OR [95% CI]	*P*	OR [95% CI]	*P*	OR [95% CI]	*P*	OR [95% CI]	*P*	OR [95% CI]	*P*
PCR-RFLP*	4	**0.626 [0.453-0.865]**	**0.004**	**0.787 [0.637-0.972]**	**0.026**	**0.726 [0.590-0.893]**	**0.002**	**0.753 [0.581-0.976]**	**0.032**	0.986 [0.835-1.164]	0.866	**0.795 [0.681-0.928]**	**0.004**
TaqMan	1	1.015 [0.786-1.311]	0.908	1.132 [0.887-1.445]	0.320	1.082 [0.858-1.364]	0.505	0.924 [0.775-1.102]	0.379	0.893 [0.755-1.056]	0.185	0.984 [0.872-1.111]	0.798
China	4	0.715 [0.477-1.072]	0.105	0.921 [0.779-1.089]	0.334	0.796 [0.584-1.048]	0.148	0.813 [0.641-1.031]	0.088	0.935 [0.828-1.056]	0.278	0.849 [0.706-1.021]	0.083
India	1	0.684 [0.358-1.309]	0.252	0.913 [0.549-1.516]	0.724	0.830 [0.527-1.305]	0.419	0.709 [0.382-1.315]	0.276	1.000 [0.617-1.620]	1.000	0.809 [0.574-1.138]	0.223
MiRNA-499 rs3746444		CC vs. TT		CT vs. TT		CT+CC vs. TT		CC vs. CT+TT		TT+CC vs. CT		C vs. T	
PCR-RFLP*	2	0.843 [0.497-1.430]	0.527	1.326 [0.555-3.164]	0.525	1.258 [0.587-2.696]	0.555	0.893 [0.598-1.333]	0.579	0.721 [0.347-1.496]	0.379	1.159 [0.733-1.832]	0.528
TaqMan	1	1.081 [0.648-1.803]	0.765	0.943 [0.776-1.145]	0.552	0.956 [0.792-1.152]	0.634	1.098 [0.660-1.827]	0.720	1.064 [0.877-1.292]	0.531	0.975 [0.827-1.149]	0.762
China	2	1.028 [0.661-1.600]	0.902	1.337 [0.650-2.748]	0.430	1.278 [0.695-2.349]	0.429	0.983 [0.634-1.524]	0.939	0.746 [0.359-1.549]	0.432	1.165 [0.789-1.720]	0.443
India	1	0.819 [0.424-1.582]	0.552	0.806 [0.398-1.630]	0.548	0.814 [0.433-1.529]	0.522	0.948 [0.602-1.492]	0.817	1.063 [0.654-1.727]	0.805	0.911 [0.645-1.287]	0.597
MiRNA-196a2 rs11614913		TT vs. CC		CT vs. CC		CT+TT vs. CC		TT vs. CT+CC		TT+CC vs. CT		T vs. C	
PCR-RFLP*	2	0.388 [0.076-1.970]	0.253	0.788 [0.559-1.110]	0.173	0.597 [0.261-1.365]	0.221	0.449 [0.103-1.956]	0.286	0.939 [0.711-1.239]	0.655	0.589 [0.230-1.505]	0.268
TaqMan	3	**0.762 [0.625-0.929]**	**0.007**	0.937 [0.804-1.092]	0.404	0.872 [0.755-1.008]	0.064	**0.807 [0.707-0.921]**	**0.001**	0.921 [0.818-1.037]	0.176	**0.871 [0.795-0.955]**	**0.003**
PCR-LDR*	1	0.886 [0.448-1.751]	0.727	1.178 [0.625-2.220]	0.613	1.049 [0.578-1.901]	0.876	0.791 [0.471-1.326]	0.373	0.788 [0.487-1.276]	0.333	0.915 [0.650-1.288]	0.612
China	5	**0.787 [0.672-0.921]**	0.003	0.946 [0.822-1.089]	0.439	0.884 [0.773-1.010]	0.069	**0.817 [0.724-0.923]**	0.001	0.922 [0.827-1.029]	0.148	**0.883 [0.817-0.954]**	0.002
India	1	**0.167 [0.086-0.323]**	<0.001	0.637 [0.374-1.085]	0.097	**0.389 [0.241-0.628]**	<0.001	**0.209 [0.114-0.381]**	<0.001	0.817 [0.510-1.309]	0.401	**0.362 [0.259-0.506]**	<0.001

*Note*: *PCR-RFLP* restriction fragment length polymorphism polymerase chain reaction, *PCR-LDR* polymerase chain reaction-ligase detection reaction

#### Relationship between miRNA-499 rs3746444 and cervical cancer

The pooled analysis showed that among the six genetic models, there was no correlation between miRNA-499 rs3746444 and the risk of cervical cancer (Table 4). Subgroup analysis indicated that this correlation remained absent in different genotyping assays and countries (Table 5).

#### Relationship between miRNA-196a2 rs11614913 and cervical cancer

**Fig. 3:**
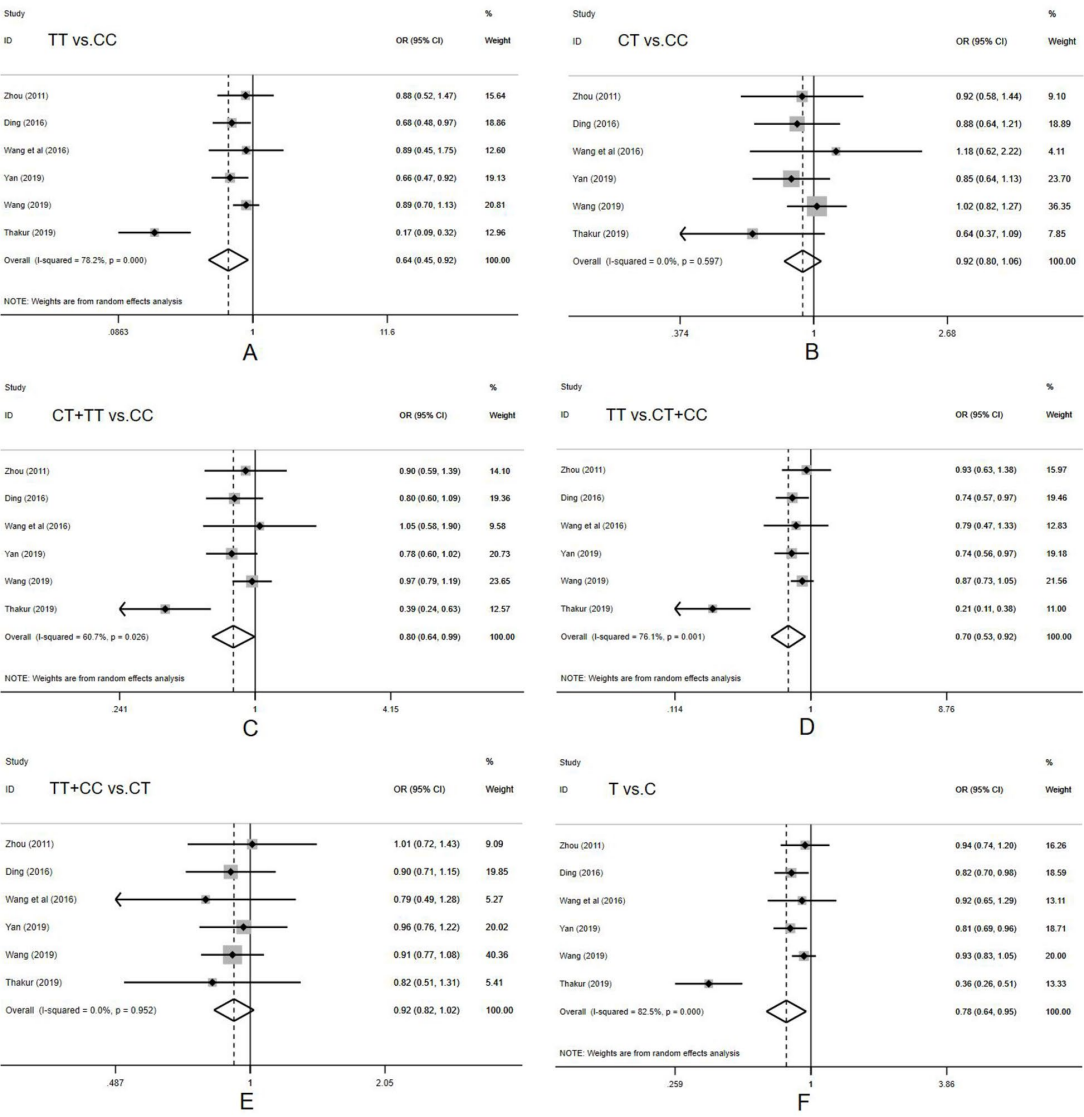
Forest plot for the association between miRNA-196a2 rs11614913 polymorphism and cervical cancer susceptibility for TT vs. CC (**A**), CT vs. CC (**B**), CT+TT vs. CC (**C**), TT vs. CT+CC (**D**), CC+TT vs. CT (**E**), and T vs. C (**F**)

The combined analysis showed that mutant TT was significantly associated with a lower risk of cervical cancer compared to the wild homozygous CC (OR = 0.641, 95% CI = 0.447-0.919, *P* = 0.016; TT vs. CC). The mutant gene T significantly reduced the risk of cervical cancer in both dominant (OR = 0.795, 95% CI = 0.636-0.994, *P* = 0.045; CT+TT vs. CC) and recessive (OR = 0.698, 95% CI = 0.532-0.917, *P* = 0.01; TT vs. CC+CT) genetic models. In the allele model, T significantly reduced the risk of cervical cancer as compared to C (OR = 0.783, 95% CI = 0.643-0.954, *P* = 0.015; T vs. C) (Table 4 and Fig. 3).

Subgroup analysis by genotyping method and country in the study of genotype analysis using TaqMan showed that the polymorphism of miRNA-196a2 rs11614913 significantly reduced the risk of cervical cancer in homozygous (OR = 0.762, 95% CI = 0.625-0.929, *P* = 0.007; TT vs. CC), recessive (OR = 0.807, 95% CI = 0.707-0.921, *P* = 0.001; TT vs. CT+CC), and allele (OR = 0.871, 95% CI = 0.795-0.955, *P* = 0.003; T vs. C) genetic models (Table 5). Studies in China showed that the polymorphism of miRNA-196a2 rs11614913 significantly reduces the risk of cervical cancer in homozygous (OR = 0.787, 95% CI = 0.672-0.921, *P* = 0.003; TT vs. CC), recessive (OR = 0.817, 95% CI = 0.724-0.923, *P* = 0.001; TT vs. CT+CC), and allele (OR = 0.883, 95% CI = 0.817-0.954, *P* = 0.002; T vs. C) genetic models. Furthermore, studies in India have shown that the risk of cervical cancer is significantly reduced in homozygous (OR = 0.167; 95% CI = 0.086-0.323; *P* < 0.001; TT vs. CC), dominant (OR = 0.389; 95% CI = 0.241-0.628; *P* < 0.001; CT+TT vs.CC), recessive (OR = 0.209; 95% CI = 0.114-0.381; *P* < 0.001; TT vs. CT+CC), and allele (OR = 0.362; 95% CI = 0.259-0.506; *P* < 0.001; T vs. C) genetic models.

### Heterogeneity

MiRNA-146a rs2910164 showed no heterogeneity in the super-dominant genetic model, and heterogeneity in the homozygote and the dominant genetic models. The results of subgroup analysis showed that the heterogeneity decreased in the studies using the PCR-RFLP analysis. No heterogeneity was observed in the homozygous and recessive genetic models for miRNA-499 rs3746444, but considerable heterogeneity was observed in the remaining models. The heterogeneity persisted after subgroup analysis. MiRNA-196a2 rs11614913 showed no heterogeneity in heterozygote and hyper-dominant genetic models, but it showed heterogeneity in the homozygote, recessive, and allele models. Subgroup analysis showed that heterogeneity decreased in studies using TaqMan analysis (Table 4).

### Sensitivity analysis

In order to explore whether the meta-analysis results were stable, sensitivity analysis was conducted. The meta-analysis results appeared to be stable.

### Publication bias

Potential publication bias of this meta-analysis was tested by Begg’s test and Egger’s test (Table 4). The results shown in the table indicate that no publication bias existed among the polymorphisms of miRNA-146a rs2910164, miRNA-499 rs3746444, and miRNA-196a2 rs11614913 in all genetic models.

## Discussion

Research on the impact of single nucleotide polymorphisms (SNPs) on the occurrence and development of diseases has increased over the years. With the development of molecular biology, discussions on genetic problems have become necessary. In the face of multiple pathogenic factors such as HPV infection, risky living environment, and host factors, which together lead to the occurrence of cervical cancer [2], while studying this process in depth, we found that the microenvironment formed by the regulation of certain genes in the human body seems to guide the occurrence of cervical cancer [26–28].

MiRNAs regulate gene expression at the post-transcriptional level, and participate in the regulation of various cellular functions. They affect cell proliferation, apoptosis, and carcinogenesis [29]. Variation of a certain miRNA, such as the conversion, insertion, deletion, or mismatch of a single base, can cause the polymorphism of the whole DNA sequence. This may in turn affect the function of cells and tissues and the whole host microenvironment [30]. Therefore, miRNAs may be used to predict the occurrence and development of cervical cancer [27]. We selected three kinds of miRNA polymorphisms (miRNA-146a rs2910164, miRNA-499 rs3746444, and miRNA-196a2 rs11614913) that have been studied more frequently in order to analyze their relationship with susceptibility to cervical cancer [16–23]. The results showed that the polymorphisms of miRNA-146a rs2910164 (mutant C allele) and miRNA-196a2 rs11614913 (mutant T allele) significantly reduced the risk of cervical cancer. There was no significant correlation between the polymorphisms of miRNA-499 rs3746444 and cervical cancer.

In the study using PCR-RFLP analysis, the polymorphism of miRNA-146a rs2910164 significantly reduced the risk of cervical cancer in homozygote, heterozygote, dominant, recessive, and allele models. With regard to the TaqMan analysis of the genotypes, the polymorphism of miRNA-196a2 rs11614913 significantly reduced the risk of cervical cancer in homozygous, recessive, and allelic models. According to the Chinese studies, miRNA-196a2 rs11614913 reduced cervical cancer risk in homozygous, recessive, and allelic models [16–22], whereas in Indian studies, it significantly reduced cervical cancer risk in homozygous, dominant, recessive, and allelic models [23]. Thus, using different genotype analysis methods and in different countries, the correlation between the polymorphisms of miRNA-146a rs2910164 and miRNA-196a2 rs11614913 and cervical cancer is somehow different. However, it cannot be denied that they can both reduce the risk of cervical cancer.

Meanwhile, a study by Yan et al. showed that the mutant T allele in the miRNA-126 rs4636297 polymorphism was associated with cervical cancer susceptibility, and the mutant T gene increased cervical cancer susceptibility compared to the wild C gene [21]. Wang et al. reported that the mutant C allele in the polymorphism of miRNA-149 rs2292832 was associated with cervical cancer susceptibility, and the mutant C gene increased cervical cancer susceptibility relative to the original T gene [22]. Furthermore, Chuntao noted that mutation of A allele to G allele in the polymorphism of miRNA-30c rs928508 could reduce the susceptibility to cervical cancer [20]. Fu K showed that miRNA-125 can negatively regulate the expression of VEGF in cervical cancer tissues and inhibit the PI3K/AKT signaling pathway to inhibit cell proliferation, invasion, and metastasis in cervical cancer, thus preventing the progression of cervical cancer [31]. The study of Lu X showed that miRNA-186-3p could inhibit the proliferation and migration of cervical cancer cell lines by inhibiting the expression of IGF1, and induce the apoptosis rate of cervical cancer cells [32]. Zhou H’s study showed that Mir-183-5p could mediate cirC_0018289 in vitro to regulate the development and angiogenesis of cervical cancer. Meanwhile, TMED5 plays a tumor-promoting role in the malignant development of cervical cancer by activating the Wnt7b/β-catenin signaling pathway, while Mir-183-5p can directly target TMED5 and inhibit its expression, so as to inhibit the occurrence of cervical cancer [33]. The above studies all indicate that miRNA expression is closely related to the occurrence and development of cervical cancer. However, due to few studies on the related miRNA polymorphisms, they were not included in the meta-analysis. These loci may present more in-depth studies in the future.

Persistent infection with high-risk HPV is a major factor in the development of cervical cancer [34, 35]. However, the occurrence of cervical cancer is a multigene, multistage abnormal regulation process [36, 37]. Studies have also shown that the abnormal expression of miRNAs affects the cervical epithelial cell carcinogenesis [38, 39]. Different miRNAs regulate different biological activities; some target to regulate the activity of tumor suppressor genes to inhibit development of the cancer cells. Others accelerate the growth and metastasis of cancer cells by stimulating proto-oncogenes and metastasis genes [39]. Studies have shown that the polymorphism of miRNA-146a rs2910164 G to C leads to increased expression of miR-146a, and the mutant CC genotype is associated with reduced risk of cervical cancer [40]. As a mediator of the pro-apoptotic transcription nuclear factor kappaB, miRNA-146a affects apoptosis and hence influences the development of cancer [41]. Each cancer tissue has its specific miRNA target [42], so the miRNA-146a polymorphism may affect the occurrence and development of cervical cancer through certain pathways. MiRNA-196a2 rs11614913 is a polymorphic site in the mature miR-196a2 sequence, and its mutation expression in situ gene C, the T allele, was found to be associated with reduced risk of cervical cancer [43]. Studies have shown that C gene increases the expression of mature miR-196a2 in cervical cancer patients, and this overexpression changes the state of binding of miR-196a2 to its target genes, thereby affecting the development of cervical cancer [19]. Tracking the changes of the two genetic polymorphism loci in human may help to understand how to reduce the incidence of cervical cancer, by changing the internal human environment. Therefore, these two sites may become the targets for the prevention and treatment of cervical cancer in the future. However, the biomarkers for targeted therapy of cervical cancer are complex and diverse, which need to be fully explored. Li S et al. also obtained 6 prognostic pivotal genes by WGCNA algorithm: SLC25A5, ENO1, ANLN, RIBC2, PTTG1, and MCM5. It also provides a broader direction for the research and treatment of cervical cancer [44].

However, this meta-analysis had several limitations: (1) Due to the fewer factors controlled by relevant studies, the relationship between age, number of abortions (Abortion refers to those who terminate before 28 weeks of pregnancy and whose fetal weight is less than 1000 g. This can be discussed in two categories: induced abortion and spontaneous abortion.), and the expression of the three miRNAs in cervical cancer patients was not analyzed. (2) The heterogeneity of some polymorphisms may be due to the patient population included (i.e., clinical heterogeneity), such as different types of cervical cancer, large age span, and the presence of other genetic diseases. At the same time, heterogeneity may also come from the research itself (i.e., methodological heterogeneity), such as different testing time and methods, and different experimental instruments. However, due to the lack of available data, all factors cannot be analyzed. It also reminds us that future studies should be designed more rigorously and record more comprehensive data to analyze results. (3) This meta-analysis did not establish whether the mixed effects of genetic polymorphisms were associated with susceptibility to cervical cancer.

## Conclusion

In conclusion, this meta-analysis showed that the polymorphisms of miRNA-146a rs2910164 (mutant C allele) and miRNA-196a2 rs11614913 (mutant T allele) could reduce the susceptibility to cervical cancer.

## Supplementary Information

**Additional file 1: Table S1**: The PRISMA checklist.

**Additional file 2: Table S2**: Search strategy table (PubMed).

## Data Availability

The data included in this study are all from the retrieved literature. For details, please refer to articles 16-23, in which the data are included in this study.

## Abbreviations

SNP: Single nucleotide polymorphisms
miRNA: micro-RNA
HPV: Human papillomavirus
NCTV: non_coding_transcript_variant
CIN: Cervical intraepithelial neoplasia
HWE: Hardy-Weinberg equilibrium
OR: Odds ratio
CI: Confidence interval
HB: Hospital-based
PCR-RFLP: Restriction fragment length polymorphism polymerase chain reaction
PCR-LDR: Polymerase chain reaction-ligase detection reaction
