# Supplementary material for: Association of three micro-RNA gene polymorphisms with the risk of cervical cancer: a meta-analysis and systematic review
Source: World J Surg Oncol. 2021 Dec 16;19:346. doi: 10.1186/s12957-021-02463-4 (PMC8675500; doi:10.1186/s12957-021-02463-4)
Supplement: Supplementary file 2 — Additional file 2: Table S2: Search strategy table (PubMed). [file 12957_2021_2463_MOESM2_ESM.doc]

| Table.S2 Search strategy table (PubMed) | |
| --- | --- |
| Number | Search terms |
|  |  |
| #1 | MicroRNAs [MeSH] |
| #2 | MicroRNA [Title/Abstract] OR miRNA [Title/Abstract] OR RNA, Micro [Title/Abstract] OR Primary MicroRNA [Title/Abstract] OR pri miRNA [Title/Abstract] OR Small Temporal RNA [Title/Abstract] OR RNA, Small Temporal [Title/Abstract] OR pre-miRNA [Title/Abstract] |
| #3 | #1 OR #2 |
| #4 | Cervical Cancer [MeSH] |
| #5 | Cervical Neoplasm, Uterine [Title/Abstract] OR Neoplasm, |
|  | Uterine Cervical [Title/Abstract] OR Uterine |
|  | Cervical Neoplasm [Title/Abstract] OR Neoplasm, Cervical |
|  | [Title/Abstract] OR Cervical Neoplasm [Title/Abstract] |
|  | OR Neoplasm, Cervix [Title/Abstract] OR Cervix Neoplasm |
|  | [Title/Abstract] OR Cancer of the Uterine Cervix |
|  | [Title/Abstract] OR Uterine Cervical Cancer [Title/Abstract] |
|  | OR Cancer, Uterine Cervical [Title/Abstract] OR |
|  | Cervical Cancer, Uterine [Title/Abstract] OR Cancer of |
|  | Cervix [Title/Abstract] OR Cervix Cancer [Title/Abstract] |
| #6 | #4 OR #5 |
| #7 | #3 AND #6 |
